# Reorientation of the first signal-anchor sequence during potassium channel biogenesis at the Sec61 complex

**DOI:** 10.1042/BJ20130100

**Published:** 2013-11-08

**Authors:** Helen R. Watson, Lydia Wunderley, Tereza Andreou, Jim Warwicker, Stephen High

**Affiliations:** *Faculty of Life Sciences, University of Manchester, Michael Smith Building, Oxford Road, Manchester M13 9PT, U.K.

**Keywords:** endoplasmic reticulum, membrane protein folding, potassium channel protein of chlorella virus (Kcv channel), site-specific cross-linking, TWIK (tandem-pore weak inwardly rectifying potassium channel)-related acid-sensitive potassium channel 1 (TASK-1), BMH, bismaleimidohexane, Cys-null, cysteine-null, EndoH, endoglycosidase H, ER, endoplasmic reticulum, Kcv, potassium channel protein of chlorella virus, OPG, opsin N-glycosylation tag, TASK-1, TWIK (tandem-pore weak inwardly rectifying potassium channel)-related acid-sensitive potassium channel 1, TM, transmembrane region, TRAM, translocating chain-associated membrane protein

## Abstract

The majority of the polytopic proteins that are synthesized at the ER (endoplasmic reticulum) are integrated co-translationally via the Sec61 translocon, which provides lateral access for their hydrophobic TMs (transmembrane regions) to the phospholipid bilayer. A prolonged association between TMs of the potassium channel subunit, TASK-1 [TWIK (tandem-pore weak inwardly rectifying potassium channel)-related acid-sensitive potassium channel 1], and the Sec61 complex suggests that the ER translocon co-ordinates the folding/assembly of the TMs present in the nascent chain. The N-terminus of both TASK-1 and Kcv (potassium channel protein of chlorella virus), another potassium channel subunit of viral origin, has access to the N-glycosylation machinery located in the ER lumen, indicating that the Sec61 complex can accommodate multiple arrangements/orientations of TMs within the nascent chain, both *in vitro* and *in vivo*. Hence the ER translocon can provide the ribosome-bound nascent chain with a dynamic environment in which it can explore a range of different conformations en route to its correct transmembrane topology and final native structure.

## Abstract 2

The Sec61 translocon provides an unexpectedly flexible and dynamic environment within which transmembrane regions of nascent polypeptides can be completely reoriented during the biosynthesis of multiple-spanning membrane proteins.

## INTRODUCTION

The majority of membrane proteins synthesized at the ER (endoplasmic reticulum) are delivered as ribosome-bound nascent chains via the SRP (signal recognition particle)-dependent pathway [1], and subsequently engage the Sec61 translocon responsible for the co-translational translocation of polypeptides into and across the ER membrane [1–3]. The Sec61 translocon discriminates between hydrophobic TMs (transmembrane regions) destined for membrane integration and hydrophilic stretches destined for complete translocation [4]; with TM integration requiring their lateral exit from the translocon into the surrounding phospholipid bilayer [2,5]. In the case of polytopic, or multi-spanning, membrane proteins, several TMs must be membrane-integrated and assembled, and both the ribosome and the Sec61 complex play key roles in this process [6]. It is generally accepted that ER translocon-associated components, such as TRAM (translocating chain-associated membrane protein) and the TRAP (translocon-associated protein) complex, may facilitate membrane insertion [3,7,8].

The ER translocon is not simply a passive conduit providing access for TMs to the bilayer, but rather it actively contributes to polytopic membrane protein biogenesis by facilitating the assembly of TMs before their complete integration [8–14]. Hence the association of TMs into pairs and bundles is a well-defined stage during theoretical and experimental studies of polytopic membrane protein folding [15,16], and current models suggest that this process can occur within the context of the ER translocon, where cohorts of TMs may assemble before integration [17–19].

Early models for polytopic membrane protein biogenesis suggested that they could be viewed as a series of individual signal sequences that were dealt with sequentially, and in essence individually, by the ER translocon [17,20]. It is now apparent that such early models are over-simplistic [14], and they are also unable to account for the complete translocon-mediated reorientation of TMs [21,22] that occurs via a carefully orchestrated series of events at the Sec61 complex [6]. The influence of one TM on the fate of its neighbour [23] further underlines the potential for a ‘non-linear’ treatment of TMs by the ER translocon, and there is even evidence for the positional editing of fully integrated TMs during the assembly of membrane protein complexes [24].

The acid-sensitive, two-pore, potassium channel protein TASK-1 [TWIK (tandem-pore weak inwardly rectifying potassium channel)-related acid-sensitive potassium channel 1], or K_2P_3.1, has four TMs separated by hydrophilic loops, two of which include the pore helix and selectivity filter domains that contribute to transport selectivity in the functional dimer [25]. Regions in TMs 2 and 4, and both pore loops, have been shown to contribute to a drug-binding site in TASK-1, providing structural insights into the open form of the channel [26], whereas N-glycosylation of TASK-1 regulates the delivery of functional channels to the cell surface [27]. In the present study, we have employed TASK-1 as a model for studying polytopic membrane protein biogenesis at the ER. Site-specific cross-linking indicated that an N-terminal region of TASK-1 incorporating the first TM (TM1), a type II signal-anchor, displays a prolonged association with the Sec61 complex during its biogenesis and integration. Adduct formation with Sec61β suggested that a proportion of nascent chains acquire an inverted or ‘head-first’ orientation at the ER translocon, a hypothesis that was validated using an N-glycosylation tag as a membrane topology reporter and reproduced with Kcv (potassium channel protein of chlorella virus), a ‘minimal’ potassium channel subunit of viral origin [28,29]. In the case of the shortest TASK-1 chain analysed, we can even detect some polypeptides that are N-glycosylated on each side of TM1, suggesting that a fraction of these molecules can reorient TM1 even after the modification of a flanking region (cf. [21]). The unusual behaviour of this very short TASK-1 chain underlines the flexibility of the environment provided by the ER translocon [6]. By analysing specific TASK-1 fragments in cultured mammalian cells, we identified a cohort of polypeptides that assume an inverted topology *in vivo*, and found that this phenotype is ‘corrected’ upon synthesis of the full-length protein. These findings provide further evidence for the dynamic nature of Sec61-mediated TM integration and underline the complexity of the molecular events leading to the acquisition of a native membrane topology.

## EXPERIMENTAL

### Materials

Where possible, amino acid changes were designed to preserve the biophysical properties of the resulting mutants by maintaining the Δ*G*^pred^ values for residues located in TMs [5] or provide a side chain of comparable size in hydrophilic loops. Cys-null (cysteine-null) versions of human TASK-1 (UniProt ID O14649) and *Paramecium bursaria* chlorella virus 1 Kcv (UniProt ID Q84568) genes were made to order (GenScript), and used valine to replace cysteine residues in TMs and serine to substitute for cysteine residues in hydrophilic regions. When synthesized *in vitro*, the wild-type and Cys-null versions of TASK-1 appear to be of identical size and N-glycosylation status upon SDS/PAGE, confirming that the alterations had not grossly perturbed membrane insertion (results not shown). The locations of novel cysteine probes were also chosen to minimize any perturbation of Δ*G*^pred^ values for membrane insertion [5]. Single cysteine residues were introduced into the constructs with QuikChange™ site-directed mutagenesis (Stratagene), and N-glycosylation sites were removed and inserted using the same approach. OPG (opsin N-glycosylation tag) derivatives of both proteins were produced from cDNAs containing an in-frame fusion of the coding region for residues 1–26 of bovine opsin (UniProt ID P02699) in place of the original start codon (TASK-1) or before it (Kcv), and all mutagenesis was confirmed by DNA sequencing. All *in vitro* studies used the pTNT vector (Promega), whereas for expression in HeLaM cells, OPG–TASK-1 derivatives were cloned into pCDNA3.1+ (Invitrogen). TorsinA Myc–His_6_ [30] was used as a positive control for EndoH (endoglycosidase H) treatment of cell lysates (see Figure 5).

### *In vitro* transcription and translation

Transcription templates were generated by PCR, in most cases incorporating a C-terminal V5 tag via the reverse primer, and RNA produced as described previously [11]. Ribosome-bound integration intermediates were generated by translating the resulting transcripts in rabbit reticulocyte lysate at 30°C for 40 min in the presence of canine pancreatic microsomes and [^35^S]methionine/[^35^S]cysteine followed by treatment with 0.1 mM aurintricarboxylic acid for 10 min at 30°C, then 2.5 mM cycloheximide (cf. [10,11,13]). Alternatively, ribosome/nascent chain complexes were dissociated using 1 mM puromycin and 20 mM EDTA at 37°C for 10 min. Membrane-associated components were isolated by centrifugation through a 750 mM sucrose cushion (120000 ***g*** for 10 min at 4°C) and membrane pellets were resuspended in 110 mM potassium acetate, 20 mM Hepes and 2 mM magnesium acetate (pH 7.2).

### Cross-linking, immunoprecipitation and deglycoslation

Resuspended membrane fractions were treated with 1 mM BMH (bismaleimidohexane) cross-linker for 10 min at 30°C, quenched with 5 mM 2-mercaptoethanol for 5 min and treated with 250 μg/ml RNase A for 5 min at 37°C [13,31]. SDS was added to 1% (v/v), samples were heated to 70°C for 10 min and then processed further as described previously [31]. Antibodies used for immunoprecipitation were: mouse anti-V5 (Serotec), rabbit anti-Sec61α (Richard Zimmerman, Saarland University, Saarbrücken, Germany), rabbit anti-Sec61β and rabbit anti-TRAM (Bernhard Dobberstein, ZMBH, Heidelberg, Germany). Samples were deglycosylated using EndoH (New England Biolabs) according to the manufacturer's instructions or by adding recombinant enzyme to samples in SDS/PAGE sample buffer and incubated at 37°C overnight.

### Cell culture and DNA transfection

HeLaM cells were maintained in DMEM (Dulbecco's modified Eagle's medium) containing 10% (v/v) FBS and 2 mM L-glutamine, 0.1 mM non-essential amino acids at 37°C, 5% CO_2_. Lipofectamine™ 2000 (Invitrogen) was used for transient transfection in accordance with the manufacturer's instructions, and cells were harvested after ~18 h.

### SDS/PAGE and sample analysis

Samples were heated in SDS/PAGE sample buffer at 37°C for 30 min before electrophoresis on 12% Tris/glycine or 15% Tris/bicine gels (in Figure 4E only; cf. [32]). Gels were fixed, dried and exposed to a phosphorimaging plate, and products visualized using a FLA-3000 (Fuji). Adducts were considered authentic (cf. Table 1), where products of the same apparent size were immunoprecipitated with both the anti-V5 antibody and a serum recognizing a component of the ER translocon, i.e. Sec61α, Sec61β and TRAM (cf. [13]). The quantification of radiolabelled products was performed using AIDA version 3.44 software (see Figure 3C). To analyse OPG–TASK-1 products expressed in HeLaM cells, lysates were prepared directly in sample buffer and, following SDS/PAGE and Western blotting, proteins detected using antibodies against the OPG (OPG–TASK-1) or Myc (control) tag respectively with donkey anti-mouse 800 IR dye-conjugated secondary (LI-COR Biosciences) antibody. Proteins were visualized and quantified using an Odyssey LC scanner and Image Studio software (LI-COR Biosciences).

**Table 1:** Summary of TASK-1 cross-linking analysis Schematic representations of the truncation lengths are shown for each column, together with the number of residues in the chain (including the 14-residue C-terminal V5 tag). These fragments are named after the last residue before the chain was truncated. Each row summarizes data from one or more cysteine residues located within either the TM indicated, or the first P-loop (P1). Cysteine mutants that generated specific adducts with subunits of the Sec61 complex (as specified in the Experimental section) are indicated with α, β or αβ where a single adduct of the nascent with both components was detected. Some cysteine probes could not be tested with short integration intermediates (NA), some combinations were not tested (NT), and a number of combinations of probe location and longer chain lengths resulted in no discrete cross-linking products being observed (NDC).

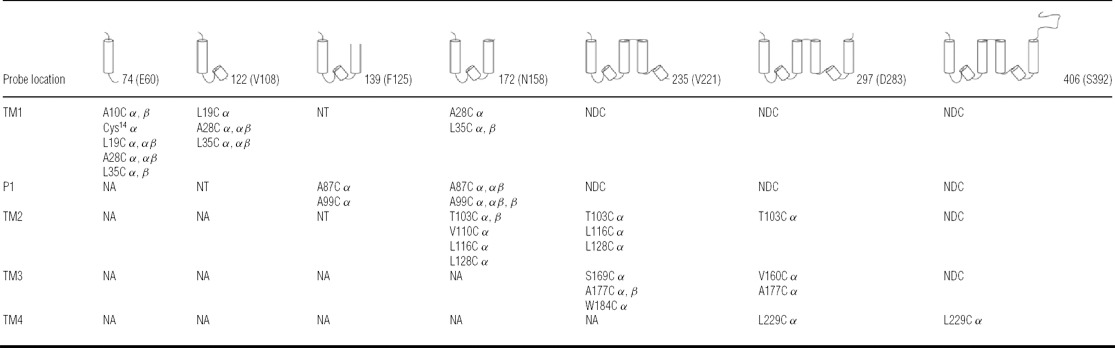

## RESULTS

Our aim was to investigate two aspects of TASK-1 biogenesis at the ER membrane: (i) the proximity of distinct TMs to the Sec61 translocon at different nascent chain lengths; and (ii) the orientation of the first TM, a type II signal-anchor, during membrane integration. To this end, we employed *in vitro* translation and site-specific cross-linking, combined with non-native N-glycosylation sites as a topology reporter, to study integration intermediates. Notwithstanding technical limitations [33], both photo- and maleimide-mediated site-specific cross-linking have provided useful insights into the components and mechanisms underlying polytopic membrane protein biogenesis at the ER (e.g. [6,8–14]). The TMs of TASK-1 each contain hydrophilic amino acids that might potentially compromise membrane insertion (Supplementary Figure S1 at http://www.biochemj.org/bj/456/bj4560297add.htm), and we have used single cysteine residues to probe their proximity to specific components of the ER translocon.

### TMs remain in close proximity to the ER translocon during TASK-1 biosynthesis

**Figure 1:**
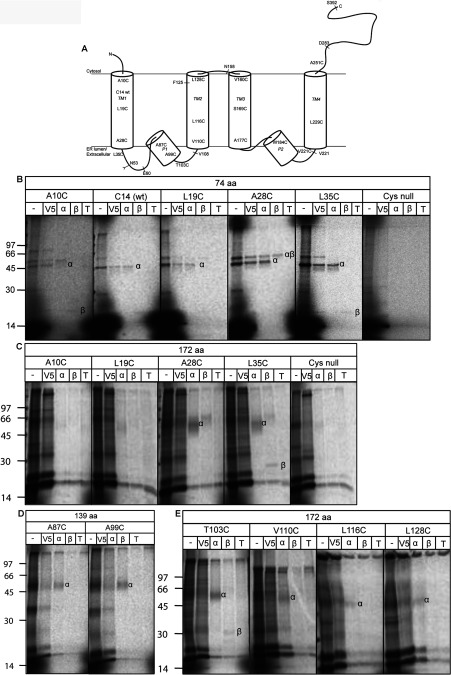
Site-specific cross-linking of TASK-1-integration intermediates (**A**) Representation of human TASK-1 showing four TMs (TM1–TM4) and two pore loops (P1 and P2) [25] (see also Supplementary Figure S1 at http://www.biochemj.org/bj/456/bj4560297add.htm). Truncated chains used to generate integration intermediates are indicated (see E60, V108, F125, N158, V221, D283 and S392, in each case a C-terminal 14-residue V5 tag was appended; see Table 1), together with locations of single cysteine targets for BMH-dependent cross-linking. The endogenous site for N-glycosylation is indicated (Asn^53^). Representative cross-linking experiments with integration intermediates of 74 (**B**), 172 (**C** and **E**) and 139 (**D**) residues (‘aa’) are shown for cysteine probes located in or near TM1 (**B** and **C**), P1 (**D**) and TM2 (**E**). Total membrane-associated products after BMH treatment (−), or products recovered via immunoprecipitation with anti-V5 (V5), anti-Sec61α (α), anti-Sec61β (β) or anti-TRAM (T) antibodies are shown. A Cys-null version of TASK-1 was used to control for the authenticity of BMH mediated cross-linking products (**B** and **C**). Adducts with Sec61α, Sec61β and Sec61α+β are labelled (α, β and αβ respectively). No discrete TRAM adducts were observed. Molecular masses are indicated in kDa.

To estimate a ‘window of proximity’ to the Sec61 complex for different TMs of TASK-1, we built a series of single-cysteine mutants and generated ribosome-bound integration intermediates for each one of increasing chain length (Figure 1A). All intermediates included a C-terminal V5 tag to identify authentically truncated polypeptides and encoding mRNAs lacked stop codons to favour the formation of ribosome-bound ER translocon-associated integration intermediates [10,13]. The treatment of 74-residue-long TASK-1 integration intermediates, containing single cysteine probes at different locations, with BMH resulted in discrete cross-linking products (Figure 1B). Among these products, adducts with subunits of the Sec61 complex could be identified by immunoprecipitation using validated antibodies known to recognize specific components of the ER translocon [10,11,13,31,34]. The most prominent cross-linking partner that could be identified in this fashion was the Sec61α subunit (Figure 1B), although we also observed a trimeric adduct of TASK-1–Sec61α–Sec61β when the cysteine probe was located at residue 28 (Figure 1B, A28C panel). A TASK-1 Cys-null variant generated no such products (Figure 1B, Cys null panel), confirming the site-specific nature of these adducts. Furthermore, adduct formation depended on a stable ribosome-bound nascent chain, hence puromycin treatment before BMH addition abolished cross-linking to components of the ER translocon (Supplementary Figure S2 at http://www.biochemj.org/bj/456/bj4560297add.htm). Taken together, these data suggest that TM1 of a 74-residue-long TASK-1 integration intermediate engages the Sec61 complex upon its delivery to the ER membrane.

Interestingly, at this shortest chain length, we also observed rather weak cross-linking products formed between a single cysteine probe located at both the N- and C-terminal side of TM1 and the single cytoplasmically located cysteine residue of Sec61β (Figure 1B, A10C and L35C panels, and Supplementary Figure S2, lane 3). The adduct with L35C was unexpected, and suggested that either TM1 is in a loop conformation at an early stage of biogenesis, or at least a proportion of nascent TASK-1 chains might assume an inverted topology during membrane integration. When the cross-linking partners of a longer integration intermediate were analysed, in addition to adducts with Sec61α, the L35C-mediated adduct with Sec61β was still present and, if anything, became more apparent (Figure 1C, L35C panel, and Supplementary Figure S2, lane 6). We extended these studies to look at probes at or near the first pore loop, P1, and TMs 2, 3 and 4 (Figure 1A, and Supplementary Figure S1) and could identify further adducts with Sec61α and Sec61β (Figures 1C–1E, Table 1 and Supplementary Figure S2). Although these cross-linking products became more diffuse with longer integration intermediates, as observed previously [11], a clear pattern emerged (Figure 1, Table 1 and results not shown). In short, these data indicate that: (i) TM1 remains in close proximity to the Sec61 complex after the P1 loop and TM2 are synthesized; (ii) P1 remains adjacent to Sec61 after TM2 synthesis; (iii) TM2 remains close to Sec61 when the nascent chain is extended to include TM3 and P2; and (iv) TM3 is adjacent Sec61α after TM4 synthesis. Taken together, these data are consistent with models suggesting that the ER translocon may provide an environment that facilitates the folding of polytopic membrane proteins by orchestrating the assembly of their TMs [11,12]. Furthermore, since Sec61β has only a single cysteine residue located in its cytoplasmic domain, its capacity to cross-link probe L35C from integration intermediates of 74 and 172 residues (Figures 1B and 1C, and Supplementary Figure S2) suggests that TASK-1 biogenesis may not simply involve the sequential threading of TMs in their native orientation into the ER translocon.

### Truncated TASK-1 chains assume a mixed topology in the ER membrane

**Figure 2:**
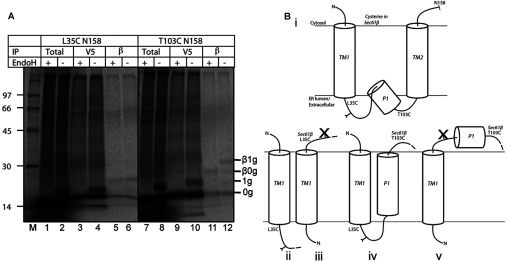
Analysis of TM1 topology by cross-linking and EndoH treatment (**A**) Integration intermediates of 172 residues (158 residues of TASK-1 plus 14-residue V5 tag) containing a single cysteine probe at either residue 35 or 103 were treated with BMH and immunoprecipitated (IP) with anti-V5 (V5) or anti-Sec61β (β) antibodies. Total products or immunoprecipitated material were then treated with EndoH (+) or were mock-treated (−). Molecular masses are indicated in kDa. (**B**) Possible conformations of TM1 as deduced from (**A**), showing the native conformation (i) and four alternatives (ii–v), together with an indication of predicted N-glycosylation status.

The ability of L35C to cross-link Sec61β in the context of two different integration intermediates suggests that the C-terminal end of TASK-TM1 may have access to the cytosolic side of the ER translocon during membrane insertion, at least in a proportion of nascent chains. To better clarify this issue, we analysed adduct formation between probes L35C and T103C and Sec61β using a stalled TASK-1 integration intermediate of 172 residues in length. Importantly, at this nascent chain length, TM1, the first pore loop (P1) and TM2 of TASK-1 should have fully exited the ribosome and hence be capable of engaging the Sec61 complex [34] (cf. Figure 1A and Supplementary Figure S1). In this case, we combined cross-linking with EndoH digestion to establish whether adducts with Sec61β were formed by nascent chains that had also been N-glycosylated at Asn^53^, and hence whether this region of the polypeptide had reached the ER lumen. In the case of the TASK-1:L35C intermediate, the Sec61β adducts were insensitive to EndoH treatment (Figure 2A, compare lanes 5 and 6) consistent with this probe cross-linking the cytosolic domain of Sec61β, and hence Asn^53^ being unable to access the oligosaccharyltransferase. Interestingly, a proportion of nascent TASK-1 chains were N-glycosylated, suggesting that, for these integration intermediates, Asn^53^ has access to the ER lumen (Figure 2A, lanes 3 and 4, compare 1g with 0g products). For comparison, the Sec61β adducts formed from a second probe location were analysed using the TASK-1:T103C mutant with a single cysteine residue located just after the P1 loop (cf. Figure 1A and Supplementary Figure S1). In this case, Sec61β adducts are EndoH-sensitive (Figure 2A, compare lanes 11 and 12, see products labelled β0g and β1g), indicating that, for this cohort of cross-linking products, Asn^53^ has access to the ER lumen whereas residue 103 can cross-link the cytosolic domain of Sec61β. Taken together, these data suggest that in at least a proportion of TASK-1 integration intermediates, TM1 has not adopted its correct topology even at a stage where P1 and TM2 have probably exited the ribosome and engaged the Sec61 complex (cf. Table 1).

### A greater proportion of TASK-1 achieves the correct topology at longer chain lengths

**Figure 3:**
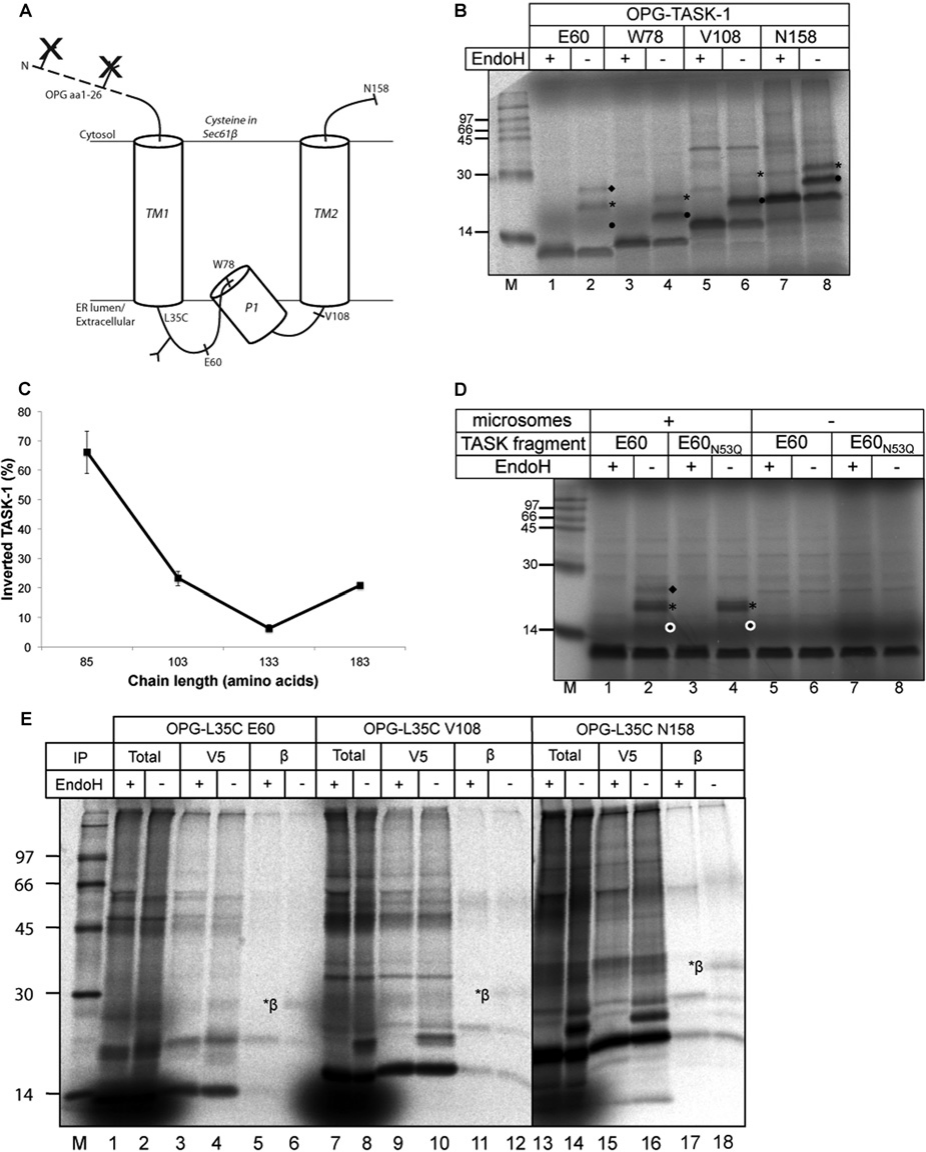
Topology analysis of OPG–TASK-1 *in vitro* (**A**) Predicted orientation of OPG–TASK-1 N-terminal region assuming the fragment assumes a native topology where the two N-glycosylation sites of OPG would not be used, but the endogenous Asn^53^ site would be modified. (**B**) A 26-residue OPG tag together with residues 2 to 60, 78, 108 or 158 of the TASK-1 coding region as indicated were synthesized in the presence of ER-derived microsomes using mRNAs with a premature stop codon at the relevant location. Resulting products were treated with EndoH (+), or were mock-treated (−), with singly (filled circle), doubly (asterisk) and triply (filled diamond) N-glycosylated species indicated. (**C**) The N-glycosylated products shown in (**B**) were quantified by phosphorimaging, and the proportion of total N-glycosylated products that had two-N-linked glycans was used to estimate the percentage of molecules where TM1 had assumed an inverted topology during the membrane integration of each OPG–TASK-1 fragment. Results are means±S.E.M. (*n*=3). (**D**) The origin of a triply N-glycosylated TASK-1 species (filled diamond) was determined by comparing the EndoH-sensitive products obtained when OPG–TASK-1 E60, and its variant OPG–TASK-1 E60_N53Q_, were synthesized in the presence and absence of ER-derived microsomes. (**E**) Ribosome-bound integration intermediates of OPG–TASK-1:L35C truncated at Glu^60^, Val^108^ or Asn^158^ of the TASK-1 coding sequence were subjected to BMH-mediated cross-linking followed by immunoprecipitation (IP) with anti-V5 (V5) or anti-Sec61β (β) antibodies. The resulting material together with a fraction of the total products were treated with EndoH where indicated (+). Adducts between doubly glycosylated TASK-1 chains and Sec61β are indicated (*β). Molecular masses are indicated in kDa in (**B**), (**D**) and (**E**).

Although our studies indicated that TM1 of at least a proportion of nascent TASK-1 chains does not adopt its final topology in a strictly sequential manner, i.e. before or even during TM2 biogenesis, the exact disposition of the N-terminus of these chains could not be clearly delineated by cross-linking alone. To this end, we incorporated an N-terminal tag comprising the first 26 residues of bovine opsin, a region of polypeptide known to be efficiently translocated when appended to either the N- or C-terminus of heterologous TMs [11,35], thus creating OPG–TASK-1. Crucially, the OPG tag includes two N-glycosylation sites that can be used as a reporter for ER translocation [31], in this case signalling TASK-1 chains where TM1 assumes an inverted type I topology (i.e. N-terminus ER-luminal; cf. Figure 3A). Our cross-linking experiments exploited truncated TASK-1 chains encoded by mRNAs lacking a stop codon, and, in order to exclude any effect from the artificial trapping of the C-terminus of the polypeptide as a ribosome-associated peptidyl-tRNA, the N-terminal glycosylation analysis was performed using truncated chains encoded by mRNAs that include a premature stop codon, thereby allowing the authentic termination of translation.

On the basis of nascent chain cross-linking to Sec61β (Figure 1 and Table 1), we focused our N-glycosylation analysis of OPG–TASK-1 on truncations that contain: TM1; TM1 and P1; and TM1, P1 and TM2. It was striking that, in every case, both singly and doubly N-glycosylated OPG–TASK-1-derived species were apparent, although their relative abundance appeared to alter with the chain length of the fragment analysed (Figure 3B, lanes 1–8, filled circles and asterisks). In the simplest scenario, OPG–TASK-1 fragments with two glycans have assumed an inverted orientation with the N-terminus in the ER lumen, whereas those with one N-linked glycan will reflect a combination of polypeptides with the correct type II topology that have been N-glycosylated on Asn^53^ (cf. Figure 2) and those with an inverted topology where only one of the two potential acceptor sites in the OPG tag is used. When the proportion of glycosylated TASK-1 species bearing two N-linked glycans was determined for each fragment analysed, we observed a measurable change with the general trend of less doubly glycosylated species at longer chain lengths (Figure 3C). In fact, following an initial decrease in the proportion of polypeptides with an inverted form of TM1, we then observed an increase with the 183-residue fragment (Figure 3C). This longest fragment incorporates the entire TM2 region of OPG–TASK-1, and this effect supports the view that TMs may act in concert during membrane integration [17–19] (and see the Discussion).

When analysing the products resulting from the *in vitro* synthesis of the shortest OPG–TASK-1 fragment, the 85-residue-long E60 truncation, we noted an additional EndoH-sensitive product migrating even more slowly than the doubly N-glycosylated species (Figure 3B, lane 2, filled diamonds). We speculated that this was most likely to be a triply N-glycosylated form of the OPG–TASK-1-E60 fragment that had been modified at both its N- and C-terminal extensions during membrane insertion (cf. Figure 3A). We confirmed this hypothesis by showing that this species disappeared when the C-terminal N-glycosylation site at Asn^53^ was mutated to glutamine (Figure 3D, compare lanes 2 and 4). Quantification showed that this triply glycosylated form of TASK-1 represented ~15±1% of the total N-glycosylated products obtained. Hence at least this fraction of molecules appears to undergo a complete inversion of TM1 at some stage during biogenesis. No evidence for triply N-glycosylated chains was apparent with the longer OPG–TASK-1 chains studied (Figure 3B). Taken as a whole, the N-glycosylation profile of these OPG–TASK-1 derivatives suggest a model where TM1 can assume at least two quite distinct topologies within the ER membrane, and suggests that the topology of TM1 may be influenced by the synthesis of additional regions of polypeptide to its C-terminus. It should be noted that this approach provides no information regarding the topology of any membrane integrated TASK-1 fragments that are not N-glycosylated (see Figure 5).

To confirm that the BMH-mediated cross-linking to Sec61β from TASK-1:L35C, where the probe is located C-terminal to TM1, and N-glycosylation of an OPG tag placed N-terminal to TM1 both reflect the same population of nascent chains, OPG–TASK-1:L35C was created and its cross-linking to components of the Sec61 translocon was analysed, paying particular attention to whether Sec61β adducts were EndoH-sensitive (Figure 3E). For the three chain lengths analysed, a substantial proportion of the Sec61β adducts are formed with doubly N-glycosylated TASK-1 nascent chains (Figure 3E, compare lanes 5, 6, 11, 12, 17 and 18). Together, these data suggest that a proportion of nascent TASK-1 polypeptides, with chain lengths up to and including 172 residues, are oriented with their N-terminus in the ER lumen and residue 35 at or close to the cytoplasmic side of the ER translocon, and hence able to cross-link Sec61β.

### TM1 of the small potassium channel Kcv displays a mixed topology

**Figure 4:**
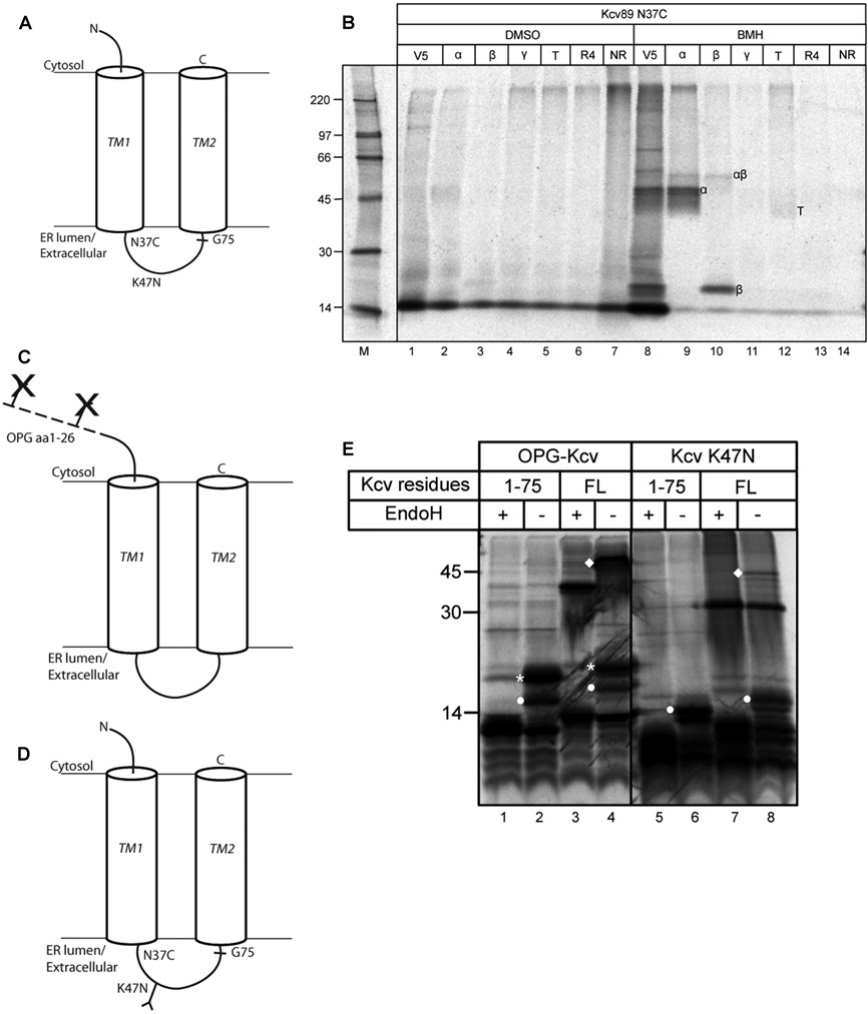
Cross-linking and topology analysis of Kcv (**A**) Schematic representation of Kcv indicating the N37C probe, the engineered N-glycosylation site K47N and the last amino acid of the Kcv coding region (Gly^75^) present in the 89-residue-long V5-tagged integration intermediate used for cross-linking. (**B**) Integration intermediates of Kcv:N37C truncated at Gly^75^ and with a V5 tag appended were treated with solvent only (DMSO) or BMH as indicated, and products were recovered by immunoprecipitation using antibodies recognizing the V5 tag (V5), Sec61α (α), Sec61β (β), Sec61γ (γ), TRAM (T), RAMP4 (ribosome-associated membrane protein 4) (R4) or FLAG as a non-related control (NR). Rabbit anti-Sec61γ and anti-RAMP4 antibodies are as described previously [40,41]. Adducts are labelled as described in the legend for Figure 1, except that T indicates an adduct with TRAM. (**C** and **D**) Schematic diagrams of the OPG–Kcv and Kcv:K47N derivatives respectively, used to study membrane topology. (**E**) OPG–Kcv or Kcv:K47N was synthesized as a 75-residue N-terminal fragment created by introducing an artificial stop codon (1–75), or the full-length protein (FL), reaction mixtures were incubated with puromycin, and the resulting products treated with EndoH (+) or left untreated (−). Doubly (white asterisk) and singly (filled white circle) N-glycosylated polypeptides are indicated. The filled white diamond indicates SDS-resistant Kcv-derived complexes that are most likely to be tetramers [29]. Whereas the samples shown in (**E**) were all resolved on the same gel, intervening lanes have been removed between lanes 4 and 5 of the final image. Molecular masses are indicated in (**B**) and (**E**).

Although previous studies have shown an initial ‘head-first’, and hence inverted, insertion of signal-anchor sequences [6,22], the suggestion that TM1 of TASK-1 can assume a completely inverted topology was nevertheless unanticipated. To establish whether this process reflected a specialized pathway or a more general feature of membrane protein biogenesis, we analysed an even simpler potassium channel subunit with only two TMs separated by a putative pore loop, the viral Kcv protein [28] (Figure 4A, and Supplementary Figure S3 at http://www.biochemj.org/bj/456/bj4560297add.htm). Preliminary studies revealed that a number of BMH-dependent adducts could be detected using a ribosome-bound integration intermediate of Kcv that contained a single cysteine probe, but not with the equivalent Cys-null version of Kcv (Supplementary Figure S4 at http://www.biochemj.org/bj/456/bj4560297add.htm). This 89-amino-acid-long integration intermediate, denoted Kcv:N37C, lacks TM2, and consists of the first 75 residues of the coding region followed by a V5 epitope tag (cf. Figure 4A). When these BMH-dependent adducts were analysed further by immunoprecipitation using antibodies recognizing well-defined components of the ER translocon, Sec61α and Sec61β were found to be two of the most prominent cross-linking partners that could be identified (Figure 4B, lanes 9 and 10, and Supplementary Figure S4). Although this is consistent with the insertion of the Kcv monomer via an ER translocon-mediated process, the cross-linking of this nascent chain to Sec61β is especially relevant, since if TM1 attains its native topology, Asn^37^ would be on the ER-luminal side of the membrane and hence incapable of forming such a product when replaced with a cysteine residue [13] (cf. Figure 4A).

The strength of the Sec61β adduct obtained with the Kcv integration intermediate suggested that a substantial proportion of these nascent chains may have an ‘inverted’ topology, and therefore we used N-glycosylation reporters to further explore Kcv biogenesis (cf. Figure 3A). To this end, OPG–Kcv, an N-terminally tagged Kcv derivative, and Kcv:K47N, a variant with a single novel site for N-glycosylation in the loop region joining TM1 and TM2, were created (see Figures 4C and 4D respectively). OPG–Kcv was synthesized in the presence of ER-derived microsomes, either in its full-length form or as a truncated chain that lacked TM2, in both cases using an mRNA containing a stop codon to terminate translation (cf. Figure 3B). For each of these OPG–Kcv polypeptides, both singly and, especially, doubly N-glycosylated species were observed at a level comparable with the non-glycosylated Kcv chains observed (Figure 4E, compare lanes 1–4). This pattern of N-glycosylation is consistent with a substantial proportion of nascent Kcv chains assuming an inverted topology at the ER translocon and maintaining this topology upon termination of translation. With full-length OPG–Kcv, some of the protein forms an SDS-resistant complex, probably a tetramer [29]. However, even this complex is sensitive to EndoH, suggesting that N-glycosylation of the OPG tag and an inverted TM1 topology does not preclude subunit assembly/oligomerization.

To establish whether any Kcv chains adopted a transmembrane orientation where TM1 assumed the expected type II topology, equivalent forms of Kcv:K47N were analysed, and a substantial proportion of chains were also N-glycosylated at this single site (Figures 4D and 4E, lanes 5–8). These data suggest that two populations of Kcv chains exist having either the N- or the C-terminal end of TM1 located in the ER lumen (cf. Figure 4A). Although Kcv:K47N is effectively N-glycosylated, the modified form of the full-length protein appears to be refractive to the formation of SDS-resistant tetramers when compared with the OPG–Kcv mutant (Figure 4E, compare lanes 3, 4, 7 and 8). Alternatively, the bulk of these SDS-resistant oligomers maybe made up of Kcv channels where TM1 spans the membrane with an ‘inverted’ topology, in which case the lack of N-linked glycans is consistent with the levels of N-glycosylated tetramer observed with full-length OPG-Kcv (cf. Figure 4E, lanes 4 and 8).

### TM1 of TASK-1 assumes mixed topologies when expressed in cultured mammalian cells

**Figure 5:**
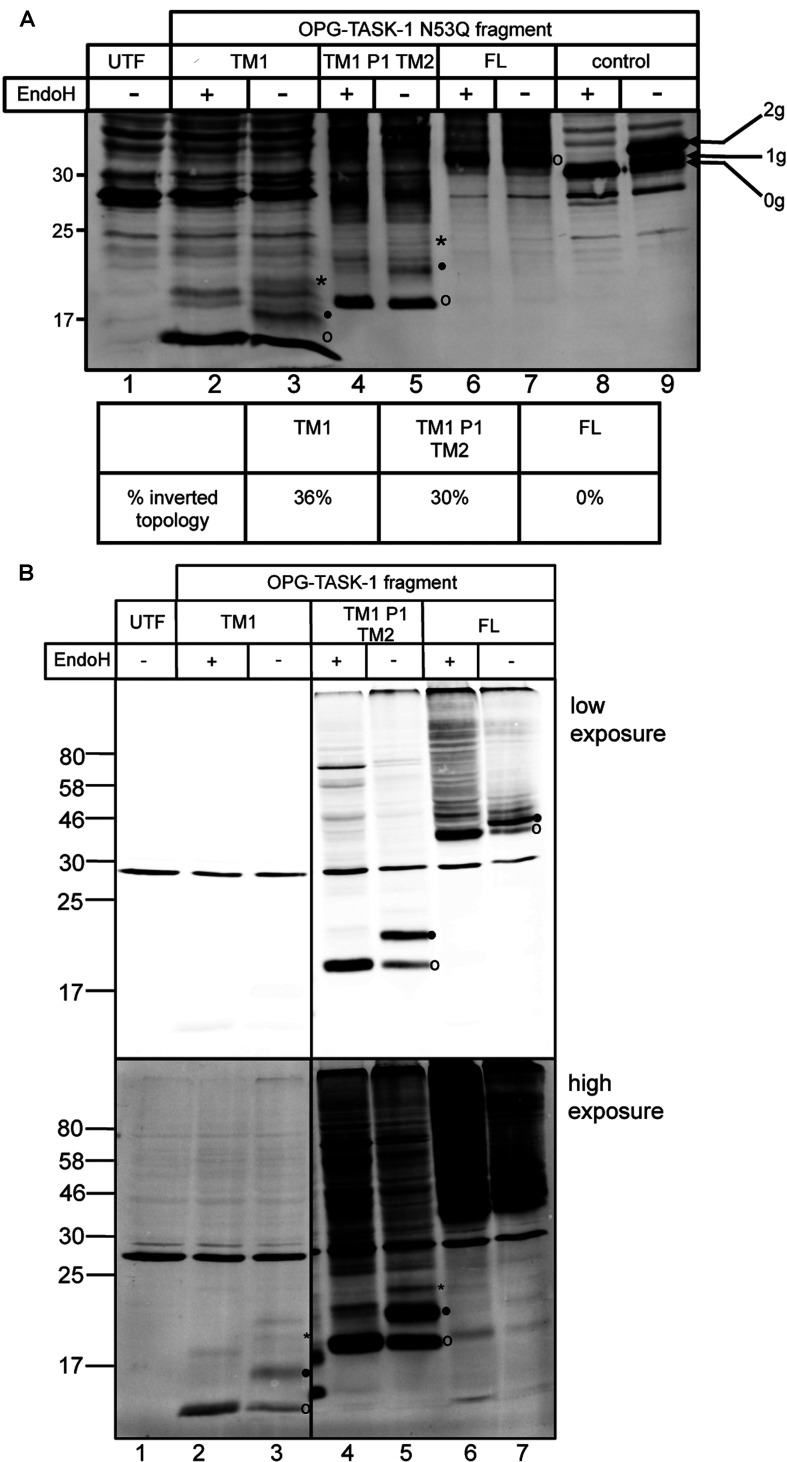
Topology analysis of OPG–TASK-1 in cultured mammalian cells (**A**) Full-length OPG-tagged TASK-1:N53Q, or N-terminal fragments thereof, were transiently transfected into HeLaM cells and products were analysed before (−) and after (+) treatment with EndoH. Untransfected cells (UTF) were included as a control. The percentage of inverted TASK-1 was determined by quantitative Western blotting of glycosylated and non-glycosylated products. (**B**) Opsin-tagged fragments or full-length (FL) TASK-1 containing the endogenous glycosylation site at Asn^53^ were analysed as described for (**A**). Both high and low exposures are shown to accommodate low steady-state levels of the smallest OPG–TASK-1-derived fragment (TM1). Open circles indicate non-glycosylated material, closed circles indicate singly glycosylated TASK-1 species, and asterisks indicate doubly glycosylated TASK-1 species. The samples shown in (**B**) were resolved on the same gel, but intervening lanes between lanes 3 and 4 have been removed from the final image. Molecular masses are indicated in kDa.

Our *in vitro* data suggested that TASK-1-TM1 had an increased tendency to assume an incorrect transmembrane topology when short fragments of the polypeptide were synthesized, whereas the native topology of TM1 was generally favoured as longer fragments of the protein were made (Figures 3B and 3C). To establish whether this inverted topology was simply a consequence of the *in vitro* system we had exploited, we investigated whether TASK-1 chains can assume a non-native topology when expressed in cultured mammalian cells. When full-length or truncated forms of OPG–TASK-1:N53Q were expressed in HeLaM cells, an ER-localized pool was observed in each case, consistent with their efficient targeting and membrane integration (Supplementary Figure S5 at http://www.biochemj.org/bj/456/bj4560297add.htm). We found detectable levels of N-glycosylated TASK-1 polypeptides modified on their N-terminal OPG tags, using fragments incorporating TM1 alone, and TM1-P1-TM2, but not with the full-length protein (Figure 5A, compare lanes 2–7). As for OPG–Kcv (cf. Figure 4D), the proportion of singly glycosylated OPG–TASK-1:N53Q at the shortest chain length analysed appeared higher than that typically observed with the OPG tag [11]. Nevertheless, since the only potential N-glycosylation sites present in these chains are N-terminal to TASK-1-TM1, both the singly and doubly glycosylated species reflect an inverted topology. Using the proportion of N-glycosylated precursors to quantify the percentage of truncated polypeptides with an inverted topology, we estimate that up to one-third of the proteins detectable at steady state are mis-inserted (Figure 5A,% inverted topology).

When equivalent fragments of OPG–TASK-1 that maintain Asn^53^ were expressed, higher levels of the singly N-glycosylated proteins were observed for the truncated fragments, consistent with a substantial proportion of these chains having the expected type II transmembrane topology (Figures 5B, lanes 3 and 5, filled circles). For full-length OPG–TASK-1, the bulk of the protein is N-glycosylated (Figure 5B, low exposure, lanes 6 and 7), in contrast with OPG–TASK-1:N53Q where no modification of the full-length precursor was detectable (cf. Figure 5A, lanes 6 and 7). On this basis, most of the full-length TASK-1 detectable at steady state has TM1 oriented with a native topology. In summary, even under circumstances where aberrant and misfolded precursors are potential targets for ER-associated degradation, we observe patterns of N-glycosylation that strongly support a model where TM1 can assume an inverted topology during TASK-1 biogenesis at the ER, and we conclude that this phenomenon is physiologically relevant to *bona fide* membrane protein synthesis *in vivo*.

## DISCUSSION

We have used *in vitro* translation, cross-linking and N-glycosylation to investigate the biogenesis of two distinct potassium channels at the ER membrane. Our key findings are: (i) the TMs of TASK-1 remain in close proximity to the Sec61 complex for a substantial period during its biogenesis, consistent with their co-ordinated assembly; (ii) TM1 of TASK-1 and Kcv can assume an inverted topology in a cohort of nascent chains during protein biogenesis at the ER; and (iii) the Sec61 translocon can accommodate at least two distinct transmembrane orientations of TASK-TM1 and Kcv-TM1. Taken together, our data are consistent with a model where the first TMs of nascent TASK-1 and Kcv chains do not adopt a stable transmembrane topology immediately upon engaging the ER translocon, but are retained in a Sec61-dependent environment that enables their subsequent reorientation in order to achieve a native topology.

Our data strongly support previous studies [6,22], both of which provide compelling evidence for a pathway involving the ‘head-first’ insertion of model signal-anchor sequences followed by their subsequent inversion to achieve their native type II topology (N-terminus cytosolic). Our work underlines the generality of a ‘head-first’ membrane insertion pathway at the ER, and shows it operates both *in vitro* and in cultured mammalian cells. Studies with a synthetic signal-anchor found both its intrinsic hydrophobicity and the ‘positive-inside rule’ influence its rate of inversion [22], and there is compelling evidence that signal-anchor reorientation is carefully orchestrated event that occurs within an environment created by both the ribosome and the ER translocon [6].

**Figure 6:**
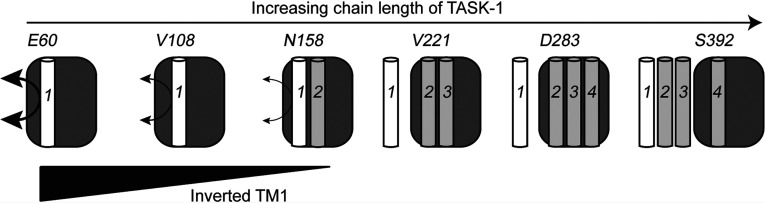
Membrane integration of TASK-1-TM1 at the ER translocon When the ribosome-bound nascent TASK-1 chain is comparatively short (exemplified here by E60), a substantial proportion of the first transmembrane domain (labelled 1) is detected with an inverted topology, consistent with these integration intermediates providing a flexible environment that is compatible with both mixed topologies and/or TM reorientation (thick double-headed arrow). On the basis of our cross-linking studies, we conclude that this environment is provided at least in part by the Sec61 translocon (dark grey box). Recent work suggests that the ribosome (not shown for simplicity) is also likely to make an important contribution [6]. As more of the nascent TASK-1 chain is synthesized (cf. V108–S392), an increasing proportion of TM1 is found to adopt a native transmembrane topology.

Our cross-linking studies suggest that, for nascent chains of both TASK-1 and Kcv, their TM1 remains associated with the Sec61 complex for a comparatively extensive period of biogenesis, a conclusion that is supported by a delay in TM exit from the ER translocon that was recently observed using an elegant FRET-based approach [14]. Furthermore, for TASK-1, we confirmed TM1 cross-linking to Sec61β from two different conformations/orientations of nascent chain, whereas nascent Kcv chains displayed unusually strong adducts with Sec61β, indicating that a substantial proportion of integration intermediates adopt an inverted topology. N-glycosylation reporters confirmed mixed topologies for both TASK-1 fragments and full-length Kcv. In the case of the shortest TASK-1 fragment studied, we could even detect a fraction of molecules that were triply N-glycosylated. This strongly suggests that at least a subset of TASK-1 chains can assume both a type I and type II transmembrane orientation during their integration into the ER membrane. Overall, the *in vitro* synthesis of longer TASK-1 fragments typically favoured a native type II topology of TM1 at the expense of the inverted form (cf. Figure 6). Importantly, this effect did not rely on the use of ribosome-tethered precursors that might favour N-terminal translocation (the present study, and [6]), and was also observed following the *in vivo* expression of TASK-1 derivatives in mammalian cells.

What is the significance of the increasing fidelity of TM1 topology as more of the TASK-1 polypeptide is synthesized? We favour a model where TM1 of the nascent TASK-1 chain transiently resides in a Sec61-enabled dynamic equilibrium such that the synthesis of additional polypeptide promotes the acquisition of its native orientation (Figure 6). In this scenario, there is a short window of opportunity during which a signal sequence can reorient within the ER translocon [21], a process that requires an appropriately assembled ribosome/translocon complex [6]. The introduction of artificial stop codons, as used in the present study, will result in the premature release of N-terminal fragments of TASK-1 from both the ribosome and the translocon, presumably resulting in a cohort of precursors where TM1 is locked in an inverted topology. Furthermore, the addition of N-linked glycans can inhibit reorientation within the ER [21], and hence the doubly N-glycosylated OPG tag we used as a reporter for exposure to the ER lumen may have acted as a ‘kinetic trap’ that stabilizes otherwise transient or reversible topological intermediates (cf. [21]). Although the cell-free system employed in the present study generates Kcv subunits that form functional potassium channels [29], we find that a substantial proportion of full-length OPG–Kcv assumes an inverted topology. Even if modification of the OPG tag accentuates this effect, our findings raise the intriguing possibility that Kcv subunits may normally acquire two different topologies, as established previously for both Ductin [36] and the Newcastle disease virus fusion protein [37].

A ‘prolonged’ association between individual signal-anchor sequences present in polytopic membrane proteins and the Sec61 complex is often assumed to reflect a role for the ER translocon in co-ordinating the assembly of TMs into pairs and/or bundles that facilitate membrane integration [10–13]. Given the complexity underlying the acquisition of a native topology by multiple different type II signal anchors (the present study, and [6,21]), it seems likely that ribosome/translocon-mediated folding events and TM inversion (cf. [6]) also contribute to these perceived ‘delays’ in the lateral exit of such TMs from the Sec61 complex. In addition to simply tethering the nascent chain in an environment where TM1 reorientation is facilitated, in the case of TASK-1 and Kcv, ongoing protein synthesis may also provide additional topological information, for example other TMs that might influence the fate of TM1 (see also [14]). Hence, in prokaryotes, the topology acquired by TM1 can be determined by later, more C-terminal, TMs [38] and in one case even a single C-terminal residue can control the membrane topology of an entire polytopic protein [39]. In summary, the once widely held view that the TMs of a polytopic protein are ‘stitched’ into the ER membrane one by one as they leave the ribosome and enter the ER translocon (cf. [17]), is giving way to compelling evidence that this event is often a much more complex and co-ordinated process [3,6,14,18,23].

## Online data

Supplementary data
