# Supplementary material for: Reorientation of the first signal-anchor sequence during potassium channel biogenesis at the Sec61 complex
Source: Biochem J. 2013 Nov 8;456(Pt 2):297–309. doi: 10.1042/BJ20130100 (PMC3898203; doi:10.1042/BJ20130100)
Supplement: Supplementary data [file bj4560297add.pdf]

BJ Cell

Biochem. J. (2013) **456**, 297–309 (Printed in Great Britain) doi:10.1042/BJ20130100

online data

SUPPLEMENTARY ONLINE DATA

Reorientation of the first signal-anchor sequence during potassium channel biogenesis at the Sec61 complex

Helen R. WATSON*[1], Lydia WUNDERLEY*, Tereza ANDREOU*, Jim WARWICKER* and Stephen HIGH*[2]

*Faculty of Life Sciences, University of Manchester, Michael Smith Building, Oxford Road, Manchester M13 9PT, U.K.

Biochemical Journal www.biochemj.org

EXPERIMENTAL

Immunofluorescence microscopy

HeLaM cells grown on glass coverslips were transfected with plasmids encoding OPG–TASK75, OPG–TASK158 or the full-length version of the protein, using Lipofectamine™ 2000 in accordance with the manufacturer's instructions. Cells were fixed and permeabilized in −20°C methanol for 5 min. Anti-opsin antibody was used to label OPG–TASK polypeptides, and anti-calnexin antibody (Sigma) was used as a marker for the endoplasmic reticulum. Alexa Fluor® 488- and 594-conjugated secondary antibodies were purchased from Jackson ImmunoResearch and DNA was stained using DAPI. Coverslips were mounted using Prolong Gold (Life Technologies) and fluorescence was visualized using a widefield Olympus BX-60 microscope with a ×60 1.40 N.A. (numerical aperture) PlanApo objective and a CoolSnap ES camera (Roper Scientific), with images captured using MetaVue software.

[1] Present address: University of Exeter Medical School, St. Lukes Campus, Magdalen Road, Exeter, Devon EX1 2LU, U.K.
[2] To whom correspondence should be addressed (email stephen.high@manchester.ac.uk).

© 2013 The Author(s)
The author(s) has paid for this article to be freely available under the terms of the Creative Commons Attribution Licence (CC-BY) (http://creativecommons.org/licenses/by/3.0/) which permits unrestricted use, distribution and reproduction in any medium, provided the original work is properly cited.

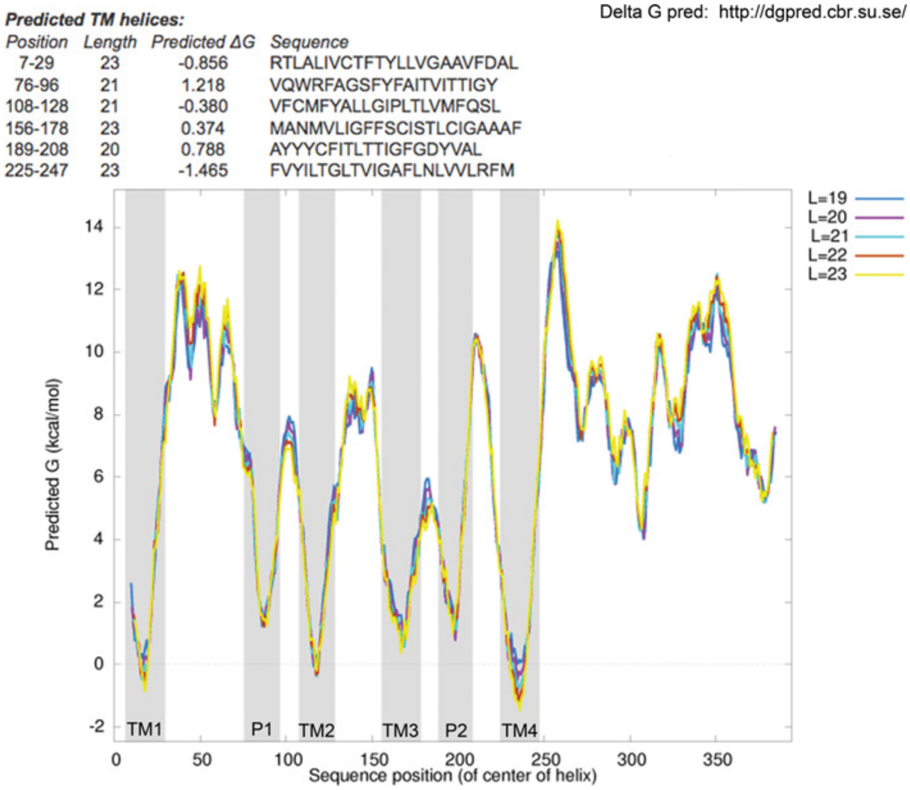

Figure S1 Transmembrane domain prediction of TASK-1

The human TASK-1 protein sequence was entered into the ΔG prediction server version 1.0 online at http://dgpred.cbr.su.se/. This predicted not only the four transmembrane domains (TM1–TM4), but also predicted the pore loops P1 and P2 as transmembrane domains. 1 kcal = 4.184 kJ.

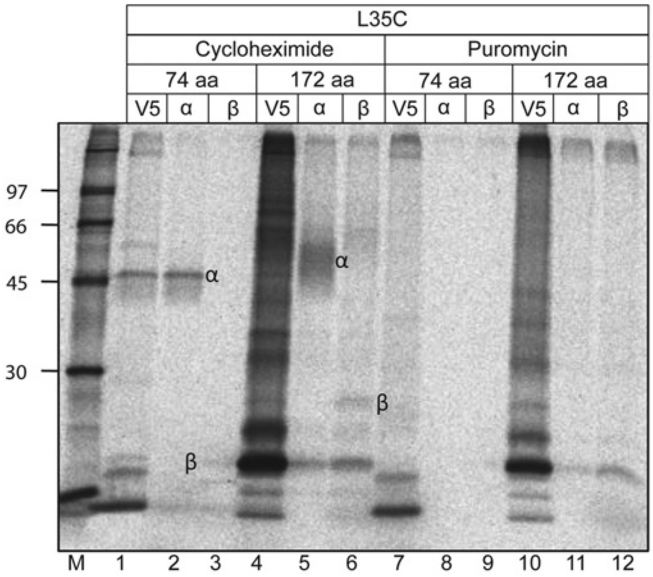

Figure S2 Cross-linking following puromycin release

TASK-1 truncations E60 and N158 lacking stop codons, both with the single cysteine mutation L35C and V5 tag were translated *in vitro*. Samples were then treated with either cycloheximide (as in all cross-linking in the present study) or puromycin (to release the nascent chain and dissociate the ribosome). BMH cross-linking was then carried out, and samples were subjected to immunoprecipitation with antibodies against the V5 tag (V5), Sec61α (α) or Sec61β (β). Adducts with Sec61α and Sec61β are indicated. Molecular masses are indicated in kDa.

© 2013 The Author(s)
The author(s) has paid for this article to be freely available under the terms of the Creative Commons Attribution Licence (CC-BY) (http://creativecommons.org/licenses/by/3.0/) which permits unrestricted use, distribution and reproduction in any medium, provided the original work is properly cited.

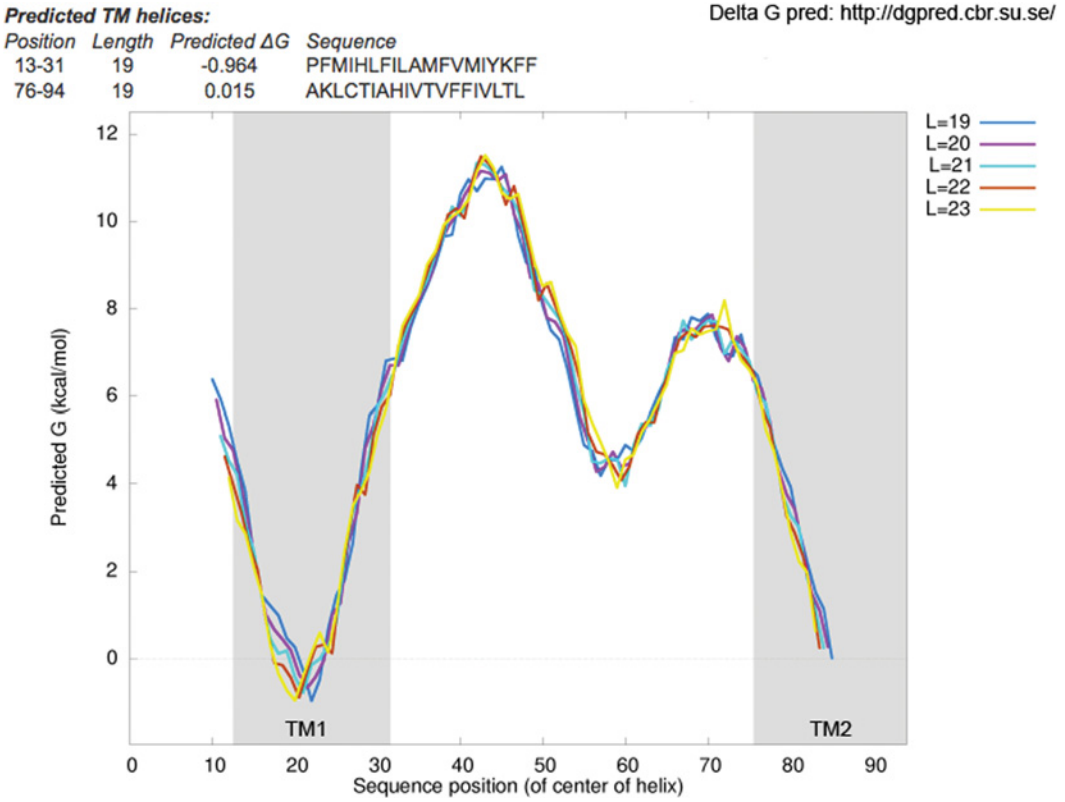

Figure S3 Transmembrane domain prediction of Kcv

The ΔG prediction server version 1.0 (online at http://dgpred.cbr.su.se/) was used to predict the location of the two transmembrane domains in Kcv. 1 kcal = 4.184 kJ.

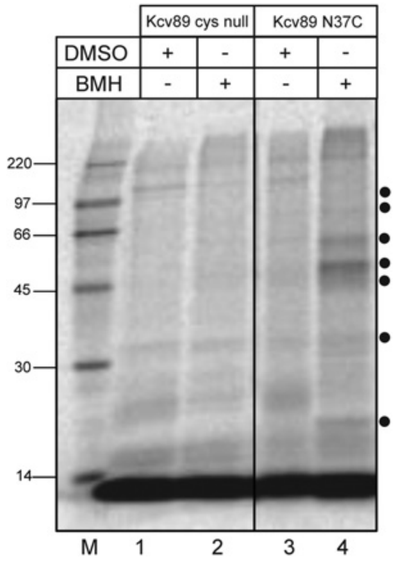

Figure S4 Cysteine-dependent adducts with Kcv membrane integration intermediates

The N-terminal 75 residues of a Cys-null form of Kcv (lanes 1 and 2), or a version with a single cysteine probe located at residue 37 (N37C, lanes 3 and 4), were synthesized using truncated mRNAs that incorporated a 14-residue V5 epitope tag at their C-termini, but lacked a stop codon. The resulting ribosome-bound peptidyl-tRNAs generated membrane-integration intermediates in the presence of ER-derived microsomes and, following isolation, these were treated with DMSO (lanes 1 and 3) or BMH (lanes 2 and 4) as indicated. A number of discrete BMH-dependent cross-linking products were observed with the N37C, but not the Cys-null variant (lane 4, filled circles). Molecular masses are indicated in kDa.

© 2013 The Author(s)
The author(s) has paid for this article to be freely available under the terms of the Creative Commons Attribution Licence (CC-BY) (http://creativecommons.org/licenses/by/3.0/) which permits unrestricted use, distribution and reproduction in any medium, provided the original work is properly cited.

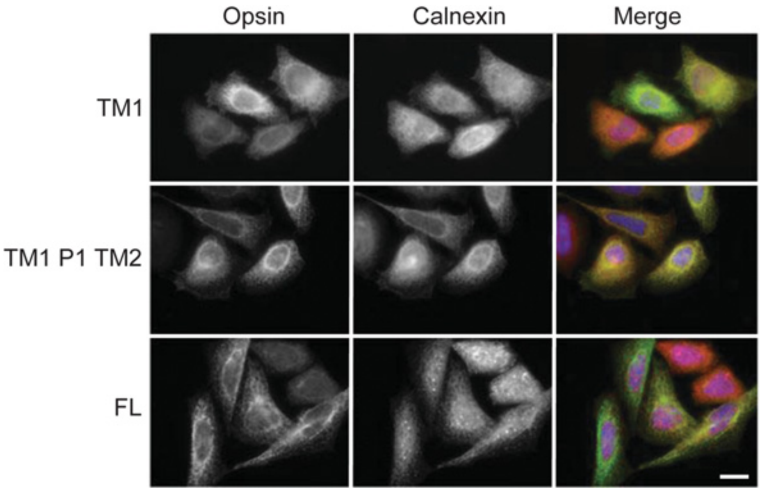

Figure S5 TASK-1-derived products localize to the ER

(**A**) HeLaM cells transiently expressing OPG-tagged fragments or full-length (FL) TASK-1 were methanol-fixed cells and stained with antibodies against the OPG tag and calnexin. Scale bars, 20 μm.

Received 17 January 2013/9 August 2013; accepted 10 September 2013
Published as BJ Immediate Publication 10 September 2013, doi:10.1042/BJ20130100

© 2013 The Author(s)
The author(s) has paid for this article to be freely available under the terms of the Creative Commons Attribution Licence (CC-BY) (http://creativecommons.org/licenses/by/3.0/) which permits unrestricted use, distribution and reproduction in any medium, provided the original work is properly cited.
